# *Aeromonas salmonicida* Infection Only Moderately Regulates Expression of Factors Contributing to Toll-Like Receptor Signaling but Massively Activates the Cellular and Humoral Branches of Innate Immunity in Rainbow Trout (*Oncorhynchus mykiss*)

**DOI:** 10.1155/2015/901015

**Published:** 2015-07-22

**Authors:** Andreas Brietzke, Tomáš Korytář, Joanna Jaros, Bernd Köllner, Tom Goldammer, Hans-Martin Seyfert, Alexander Rebl

**Affiliations:** ^1^Leibniz Institute for Farm Animal Biology (FBN), Institute of Genome Biology, Wilhelm-Stahl-Allee 2, 18196 Dummerstorf, Germany; ^2^Friedrich Loeffler Institute, Federal Research Institute for Animal Health, Institute of Immunology, Südufer 10, Insel Riems, 17493 Greifswald, Germany; ^3^Department of Pathobiology, School of Veterinary Medicine, University of Pennsylvania, 3800 Spruce Street, Philadelphia, PA 19104, USA

## Abstract

Toll-like receptors (TLRs) are known to detect a defined spectrum of microbial structures. However, the knowledge about the specificity of teleost Tlr factors for distinct pathogens is limited so far. We measured baseline expression profiles of 18 *tlr* genes and associated signaling factors in four immune-relevant tissues of rainbow trout *Oncorhynchus mykiss*. Intraperitoneal injection of a lethal dose of *Aeromonas salmonicida* subsp. *salmonicida* induced highly increased levels of cytokine mRNAs during a 72-hour postinfection (hpi) period. In contrast, only the fish-specific *tlr22a2* and the downstream factor *irak1* featured clearly increased transcript levels, while the mRNA concentrations of many other *tlr* genes decreased. Flow cytometry quantified cell trafficking after infection indicating a dramatic influx of myeloid cells into the peritoneum and a belated low level immigration of lymphoid cells. T and B lymphocytes were differentiated with RT-qPCR revealing that B lymphocytes emigrated from and T lymphocytes immigrated into head kidney. In conclusion, no specific TLR can be singled out as a dominant receptor for *A. salmonicida*. The recruitment of cellular factors of innate immunity rather than induced expression of pathogen receptors is hence of key importance for mounting a first immune defense against invading *A. salmonicida*.

## 1. Introduction

The vertebrate immune system consists of a conserved innate system complemented by a highly specialized (adaptive) immune system. Both branches of immunity communicate and collaborate in a bipartisan way to ensure the effective destruction of potentially harmful microbes [1]. Pattern recognition receptors (PRRs) are crucial germ-line encoded components of the innate branch, as they recognize directly and immediately conserved microbial structures and molecular motifs (MAMPs, microbe-associated molecular patterns, previously known as PAMPs) as well as immunogenic endogenous molecules released from the infected host (DAMPs, damage-associated molecular patterns) [2–4].

**Figure 1:**
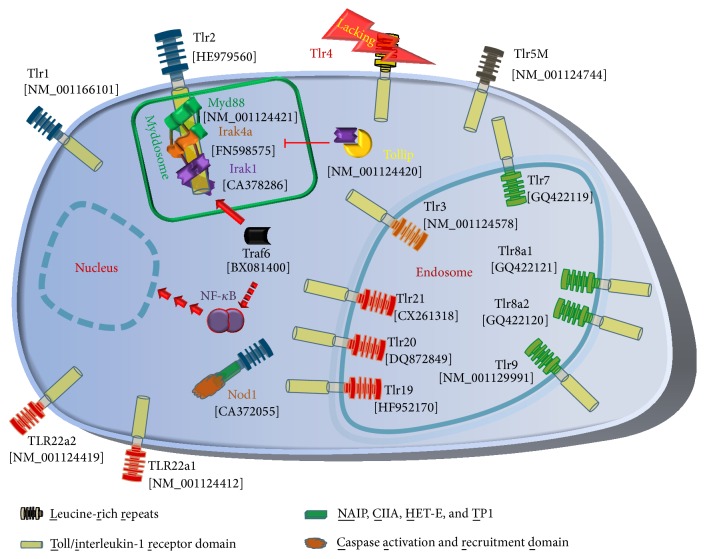
Pathogen recognition in trout. Toll-like receptors, Nod1, and downstream factors as known from trout are listed with their GenBank accession numbers. Different colors of the LRR regions factors indicate the membership to individual Tlr families (TLR1 (blue), Tlr3 (orange), Tlr5 (black), Tlr7 (green), and Tlr11 (red)). Notably, a Tlr4 ortholog is absent in salmonid fish (marked with a flash). The Myddosome consisting of Myd88, Irak4a, and Irak1 (inside the green box) binds to the activated Tlr and recruits Traf6 and further downstream factors (indicated with a broken arrow), which in turn activate NF-*κ*B. Tollip functionally inhibits Irak1 by preventing its recruitment into the Myddosome complex.

Toll-like receptors (TLRs) are the best characterized innate immune receptors. More than 20 TLRs clustered in six subfamilies have been identified in more than a dozen of fish species [5–7] (Figure 1). They provide a wide spectrum for recognizing the plethora of aquatic pathogens. Upon ligand binding, TLRs dimerize and undergo conformational changes to recruit the Myddosome to the activated toll/interleukin-1 receptor domain (TIR) [8]. In mammals, this helical structure consists of six MYD88 (myeloid differentiation primary response protein 88) adaptor molecules, onto which a layer of four IRAK4 (interleukin-1 receptor-associated kinase 4) serine/threonine kinases and another layer of four IRAK2 or IRAK1 factors are assembled [8]. The composition of teleost Myddosome is unknown so far, although functional interaction of the complex Myd88-IRAK4a with the TIR domain has been reported [9]. However, no IRAK2 factor has yet been found in any teleostean fish species [5, 6].

The activated receptor complex promotes the dissociation of IRAK1 from its functional repressor TOLLIP (toll-interacting protein) allowing its association with TRAF6 (TNF receptor-associated factor 6) and further downstream factors to activate either NF-*κ*B or interferon regulatory transcription factors or mitogen-activated protein kinases [10]. This TLR-MYD88-IRAK-TRAF6 signaling pathway is well conserved, not only in vertebrates but also in* Drosophila* [11, 12]. The activated cascade results in enhanced expression of immune factors such as cytokines provoking inflammation and allowing the communication with the adaptive branch of immunity [12].

The Gram-negative bacterium* Aeromonas salmonicida* ssp.* salmonicida* is the causative agent of furunculosis, a serious disease of salmonid fish inducing high mortality even after a low-dose intraperitoneal injection [13]. Pathogenic challenges induce not only the massive activation of proinflammatory mediators [14–17], but also enhanced transcription of* tlr*-encoding genes in fish [18–20] as reported previously for mammals [21–23]. The dominant MAMP from Gram-negative bacteria is lipopolysaccharide (LPS), known in mammals to be specifically and solely recognized by TLR4 [24]. No TLR4 ortholog has been identified so far in salmonid fish, and LPS recognition in bony fish is unclear to date [6, 25, 26]. Moreover, modulation of the expression pattern of the entire TLR panel in response to* A. salmonicida* has not been reported from rainbow trout. We therefore profiled the expression of 13* tlrs* belonging to five subfamilies of these receptors (Figure 1) in immune tissues (spleen, head kidney, liver, and thymus) from healthy and* A. salmonicida*-infected rainbow trout. Moreover, we also included other genes encoding downstream factors of TLR signaling into analysis, that is, the TLR adapter* myd88 *[27]; the key kinases* irak4a *[9] and* irak1*; the NF-*κ*B-activating factor* traf6*; and the inhibitor of TLR signaling,* tollip* [28]. This panel of candidate factors should provide a comprehensive overview of the transcriptional regulation of TLR signaling in trout during* A. salmonicida* infection.

## 2. Materials and Methods

### 2.1. Experimental Infection and Tissue Sampling

Rainbow trout (“steelhead”; Trout Lodge, Tacoma, USA) were kept in 300-l tanks at 15°C in partially recirculating water systems. The water quality was monitored daily. The light period was 12 h per day and night. Fish were fed with commercial dry pellets.

*A. salmonicida *subsp.* salmonicida *(wild type strain JF 2267) was used for experimental infection of trout. The bacteria were cultivated from cryoconserved batches (Microbank, PRO-LAB Diagnostics, Cheshire, UK) in LB broth (SIFIN) at 15°C for 72 h. The initial cultures were checked for purity by Gram-staining and observation of cell morphology. The bacterial suspension was concentrated by centrifugation (4300 rpm, 10 min, 4°C). The bacterial pellet was washed once in sterile 0.9% sodium chloride solution and diluted to 1 × 10^8^ bacteria/mL.

We injected lethal doses of* A. salmonicida *to conceivably induce uniform physiological reactions in all individual fish. One group of fish (*n* = 30) was infected by peritoneal injection with 200 *μ*L PBS (phosphate-buffered saline) containing 1 × 10^7^ *A. salmonicida* while a control group (*n* = 5) received 200 *μ*L PBS only. Five fish per group were sampled at 0-, 6-, 12-, 24-, 48-, and 72-hour postinfection (hpi). Peritoneal injection and anaesthetization of rainbow trout with phenoxyethanol prior to sampling were conducted in compliance with terms of the German Animal Welfare Act (§ 4(3) TierSchG). The experimental protocol was approved by the Animal Care Committee of the State Mecklenburg Western Pomerania (Landesamt für Landwirtschaft, Lebensmittelsicherheit und Fischerei, Mecklenburg-Vorpommern, Germany; LALLF M-V/TSD/7221.3-2.5-008/10). Tissue samples of spleen, head kidney, liver, and thymus were immediately snap-frozen and stored in liquid nitrogen.

### 2.2. RNA Isolation and cDNA Synthesis

Tissue samples were powdered in a mortar under liquid nitrogen and total RNA was subsequently extracted using QIAzol Lysis Reagent followed by purification with RNeasy Mini spin columns, as provided in the RNeasy Plus Universal Kit (Qiagen, Hilden, Germany). The RNA concentrations were determined with the NanoDrop 2000 photometer (Thermo Scientific, Waltham, MA, USA). 1.5 *μ*g total RNA from each sample was transcribed into cDNA using the Super Script II kit (Invitrogen/Life Technologies, Karlsruhe, Germany) and gene-specific antisense oligonucleotides (Table 1). Complementary DNA aliquots equivalent to an input of 75 ng total RNA were used in subsequent RT-qPCR reactions.

### 2.3. RT-qPCR

We derived rainbow trout specific oligonucleotide primer pairs to amplify cDNA sequence fragments of 13* tlr *genes (*tlr1*,* tlr2*,* tlr3*,* tlr5*,* tlr7*,* tlr8a1*,* tlr8a2*,* tlr9*,* tlr19*,* tlr20*,* tlr21*,* tlr22a1*, and* tlr22a2*); of five genes coding for downstream signaling factors of the TLR pathway (*myd88*,* irak4a*,* irak1*,* tollip*, and* traf6*); of the* nod1* gene (nucleotide-binding oligomerization domain-containing 1) representing an alternative PRR. All relevant GenBank accession codes are indicated in Figure 1. Primers for five cytokine-encoding genes (*il1b*,* tnf*,* il8*,* il10*, and* tgfb*) and two immune cell marker genes (*ighm*,* trb*) were also derived (Table 1). The oligonucleotide primers were designed using the Pyrosequencing Assay Design software v.1.0.6 (Biotage, Uppsala, Sweden). The resulting PCR products were cloned (pGEM-T Easy; Promega, Mannheim, Germany) and sequenced (Applied Biosystems 3130 Genetic Analyzer; Life Technologies) to ensure the authenticity of the respective gene fragments. Primer efficiencies are given in Table 1.

cDNA copy numbers were quantified on the LightCycler 96 System (Roche, Basel, Switzerland) using the SensiFAST SYBR No-ROX Kit (Bioline, Luckenwalde, Germany). After each RT-qPCR run, PCR products were visualized on 3-% agarose gels to validate product size and quality. Melting curve analyses evaluated the amplification of single products per sample (specific Tm values are listed in Table 1). Standard curves were generated based on 10-fold dilutions (10^3^ to 10^6^ copies) of the respective cloned fragments serving as external standards in the analytical runs. Copy numbers were calculated on the basis of linear regression of the standard curve (*R* ^2^ > 0.99 in each case).

### 2.4. Flow Cytometry

The cells in the peritoneal cavity, the site of experimental infection, were retrieved through lavage with 5 mL ice-cold PBS containing 5 mM EDTA (ethylenediaminetetraacetic acid). For the analysis of cell number and distribution of lymphoid and myeloid cell populations, 100 *μ*L cell suspension was diluted in 300 *μ*L PBS/0.01 M EDTA solution. Cells counts were acquired by FACSCalibur (Becton Dickinson, Germany) in “HIGH-throughput” mode for 20 seconds. The cell composition was analyzed using a set of monoclonal antibodies as previously described [29]. In brief, the total number of leukocytes was incubated with diluted antibodies for 30 minutes. Antibodies were either directly labeled with fluorochrome or cells were first incubated with an unlabeled specific antibody and subsequently for another 30 minutes with the corresponding mouse isotype-specific antibody, labeled either with fluorescein isothiocyanate (FITC; Rockland, Limerick, PA, USA) or R-phycoerythrin (RPE; Jackson ImmunoResearch Laboratories, West Grove, PA, USA).

### 2.5. Statistics

The data are presented as the mean ± standard error of the mean SEM. To assess statistical significances, we performed one-way analysis of variance (ANOVA) followed by Tukey's post hoc test as provided by SigmaPlot (Systat Software Inc., San Jose, CA, USA). *p* values <0.05 were considered as indicating significant differences.

## 3. Results

### 3.1. Tlrs and Associated Factors Were Most Abundantly Expressed in Spleen from Healthy Trout

**Figure 2:**
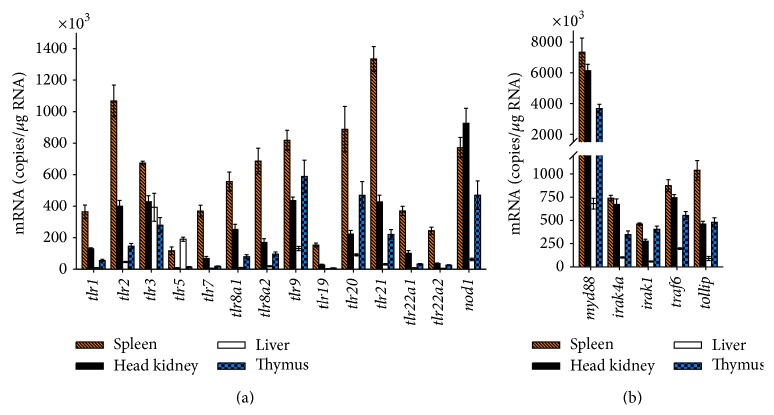
Copy number of (a) PRRs and (b) downstream signaling factors in selected tissues from healthy trout. Quantitative RT-PCR was used to determine the number of transcripts/*μ*g total RNA (ordinate) in spleen (dashed bars), head kidney (filled bars), liver (open bars), and thymus (chequered bars) of five healthy rainbow trout. Bars indicate mean ± SEM.

We performed a quantitative real-time PCR (RT-qPCR) profiling across 19 factors constituting the recognition of MAMPs in rainbow trout. Regarding the tissue-specific expression, we found that all 13* tlr* genes were significantly expressed in spleen, head kidney, liver, and thymus (Figure 2(a)). However, we observed large differences in basal levels of* tlr *transcripts. Spleen tissue featured the highest mRNA concentrations of almost all the Tlr-encoding genes, as frequently found in other fish species [26]. The copy numbers ranged from a minimum of 0.12 ± 0.02 × 10^6^ (*tlr5*) to a maximum of 1.3 ± 0.18 × 10^6^ copies (*tlr21*) per *μ*g RNA. The concentrations in head kidney amounted to approximately one-third of the values found in spleen with* tlr5* featuring the lowest abundance of only 7% of the level in spleen. Levels in liver were generally much lower than in spleen, amounting for eleven of the considered* tlr* genes on average to less than 10% of the levels as recorded in spleen. Yet,* tlr5* and* tlr3* were found to be exceptional since their mRNA concentrations reached 163% and 58% of those values measured in spleen.

We could not find distinctive tissue-specific expression profiles distinguishing the expression levels of the known transmembrane* tlr* genes (e.g.,* tlr1*,* tlr2*, and* tlr5*) from those of the known endosomal* tlr*s (*tlr3*,* tlr7*,* tlr8a1*,* tlr8a2*, and* tlr9*) or of the fish-specific* tlr*s (*tlr19*,* tlr20*,* tlr21*,* tlr22a1*, and* tlr22a2*), the latter belonging all to the TLR11 family (Figure 1). Comparing the relative expression intensities between the various* tlr* genes, we recorded the highest mRNA abundances (>220,000 transcripts/*μ*g RNA) for* tlr9*,* tlr20,* and* tlr21* in spleen, head kidney, and thymus, while* tlr5* and* tlr19* were found to be expressed on comparatively low levels in those tissues (<160,000 transcripts/*μ*g RNA).

Levels of* nod1* transcripts were recorded to monitor the expression of an alternative PRR [30].* Nod1* copy numbers were on similar levels as* tlr2*,* tlr9*, and* tlr20* in spleen and liver and exceeded in head kidney by more than twofold the level of the quite strongly transcribed* tlr9* or* tlr21*.

Transcripts encoding downstream factors of the TLR pathway were also abundant (Figure 2(b)). Copy numbers of* irak4a*,* irak1*,* traf6*, and* tollip* genes were in a similar range as the* tlr *genes.* Myd88*, in contrast, exceeded by 3- to 15-fold the level of those other factors.

### 3.2. Severe Infection with* A. salmonicida* Strongly Induced* il1b*,* tnf*, and* il8*

Rainbow trout were intraperitoneally infected with a high dosage (1 × 10^7^ cfu) of* A. salmonicida* ensuring establishment of a uniform clinical infection in all individuals. At the end of the trail, infected trout displayed classical apathetic behavior; hemorrhages in liver; enlarged spleen and liver; and swollen intestine as typical symptoms of acute infection.

To characterize the course of the up-running immune defense after infection, we profiled the expression of several cytokine-encoding genes in our four target tissues spleen, head kidney, liver, and thymus. Il1b [31, 32], Tnf [33], and Il8 [34, 35] play key roles during inflammation of rainbow trout. Il10 [36] and Tgfb [37] act as regulatory cytokines [38].

**Figure 3:**
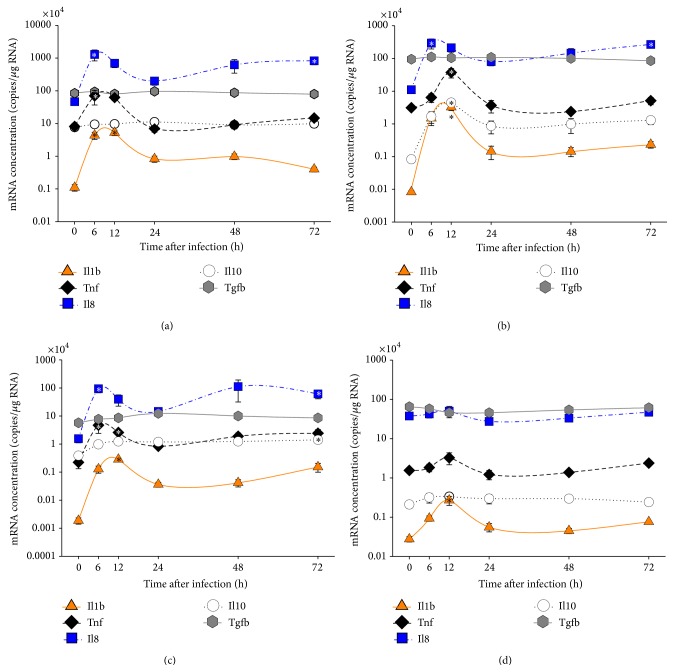
Relative levels of mRNAs encoding cytokines in selected immune organs of trout after infection with* A. salmonicida*. The relative number of mRNA copies (ordinate) encoding Il1b (triangles; full line), Tnf (diamonds; broken line), Il8 (squares; line-dot-line), Il10 (circles; dotted line), and Tgfb (hexagon; full gray line) is plotted against the time after infection (abscissa). Different tissues (a) spleen, (b) head kidney, (c) liver, and (d) thymus were collected from five individuals/time point. Values are given as mean ± SEM; the pertinent data are listed in Table 2. Note that the relative quantity is presented on a log10 scale. Asterisks indicate significant differences with *p* < 0.05 compared to the control group (0 h).

As early as 6 hpi, we found significantly elevated* il8 *transcript levels (*p* ≤ 0.03) in liver (60-fold), spleen (28-fold), and head kidney (27-fold) compared to naïve trout (Figure 3; Table 2). At 12 hpi, we recorded strongly increased* il1b* mRNA abundances (with *p* < 0.01) in head kidney (393-fold), liver (152-fold), spleen (48-fold), and thymus (10-fold) accompanied by moderately increased* tnf* mRNA levels (with *p* < 0.03) in head kidney (12-fold), liver (12-fold), and spleen (8-fold). At 72 hpi, we found a second, less pronounced upregulation of* il8* gene expression (with *p* < 0.02) in liver (39-fold), head kidney (24-fold), and spleen (18-fold).* Il10* expression was highly upregulated in head kidney at 12 hpi (54-fold) indicating the onset of immune-dampening mechanisms restricting inflammation. Only* tgfb* gene remained on a similar expression level throughout the infection.

These data together validate that the severe infection initiated strong inflammation.

### 3.3. Marginal Regulation of Factors Constituting the TLR Signaling Cascade during Severe* Aeromonas salmonicida* Infection

**Figure 4:**
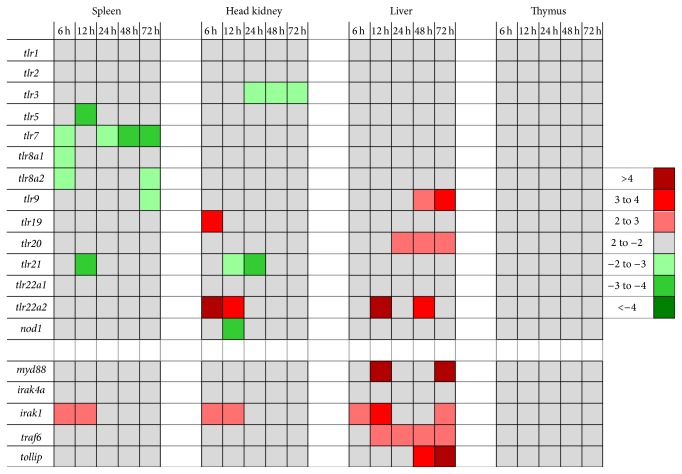
Modulation of the mRNA concentration of factors contributing to TLR signaling during infection. Colored fields represent significantly (*p* < 0.05) altered fold changes (>2-fold) of the mRNA concentrations as measured in the respective organs at the various times after infection. The pertinent data are listed in Tables 3(a) and 3(b).

A key aspect of our study was to profile the expression of all our candidate Tlr factors during* A. salmonicida* infection identifying prominently regulated members of this receptor family. The data are visualized in Figure 4 and listed in Table 3(a). Surprisingly, from the 13* tlr *genes studied, only the expression of* tlr22a2* was clearly and quickly induced in head kidney (6 hpi: 6-fold; 12 hpi: 4-fold over controls) and liver (12 and 48 hpi: 4-fold over controls) in the infected trout. The genes encoding Tlr9, -19, and -20 were induced in liver to a low extent (2- to 3-fold; *p* < 0.05). Transcript levels of* tlr1* and* tlr22a1* remained stable over time and those encoding Tlr2, -3, -5, -7, -8a1, -8a2, -9, -21, and Nod1 were even downregulated (<4-fold) in spleen or head kidney.

No downregulation was found for any of our candidate downstream factors of TLR signaling in any of the four tissues (Figure 4; Table 3(b)). Levels of all these factors remained virtually stable in thymus during the entire infection period. In contrast, all these factors were significantly upregulated in liver, at least at some time point during infection. The mRNA concentration of* myd88* was most prominently upregulated (>6-fold, 12 hpi and 72 hpi) and remained on elevated levels throughout. The* traf6* mRNA concentration rose at 12 hpi to a subsequently sustained 2-fold increased level. The mRNA concentration of* tollip* was raised at 24 hpi by ~3-fold and increased further until 72 hpi. The induction profile of* irak1* was remarkable in so far as it significantly increased >2-fold in three organs, spleen, head kidney, and liver already 6 hpi. This elevated level was sustained in liver throughout the infection period (*p* > 0.05 at 24 and 48 hpi), while it clearly dropped down later compared to 12 hpi in spleen and head kidney.

### 3.4. Infection Quickly Induced an Influx Mainly of Myeloid Cells into the Peritoneum

**Figure 5:**
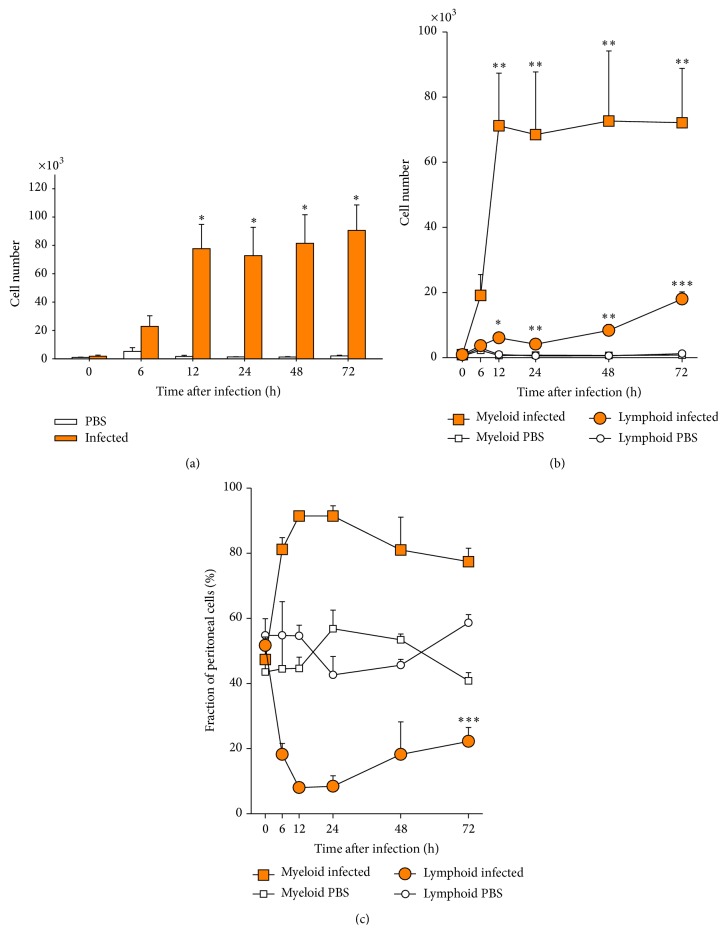
Kinetics of leukocyte recruitment into the peritoneum after infection with* A. salmonicida*. (a) The total number of leukocytes in the peritoneal fluid in infected trout (filled bars) and PBS-injected controls (open bars) was determined with flow cytometry and is given as mean ± SEM from five fish per time point (ordinate). (b) Differentiation of the number of recruited cells into myeloid cells (square symbols) and lymphocytes (circles). (c) Alteration of the percentage of myeloid cells (square symbols) and lymphocytes (circles) after PBS injection (open symbols) or infection with* A. salmonicida* (filled symbols) as calculated from the data given in (b). Asterisks denote statistical significance with *p* < 0.001 (*∗∗∗*), *p* < 0.01 (*∗∗*), and *p* < 0.05 (*∗*), compared to controls (0 hpi), assessed with one-way ANOVA, followed by Tukey's post hoc test.

Activation of the early cellular immune response in the peritoneal cavity, the site of infection, was indicated by a very strong increase in the total number of cells (Figure 5(a)). The number of peritoneal leukocytes increased during the first 12 hpi by >40-fold (*p* < 0.05). Their number remained on this high level for another 60 h in all infected fish. Only slight and statistically insignificant changes were observed in the control group injected with PBS (<2-fold, *p* = 0.6).

Flow cytometry was used to differentiate myeloid from lymphoid cells in peritoneal lavages (Figures 5(b) and 5(c)). Myeloid cells (mainly monocytes/macrophages and granulocytes) were the first to become massively recruited. Their number increased already during the first 6 hpi by 43-fold (*p* < 0.05) and reached a plateau level of a 162-fold (*p* < 0.01) increase at 12 hpi (Figure 5(b)). Significant amounts of lymphoid cells (most likely B cells; maybe T cells; natural killer-like cells) were also recruited into the peritoneum, but with a slower, yet steady rate. Their number was increased by 41-fold (*p* < 0.02) at 72 hpi. The different rates for recruiting both cell types eventually resulted in a grossly altered composition of the peritoneal cell population. The relative proportion of myeloid (44%) and lymphoid cells (55%) was quite balanced in control trout (Figure 5(c)). The* A. salmonicida* infection changed the situation tremendously. Myeloid cells constituted more than 80% of the peritoneal cells already at 6 hpi, whereas lymphoid cells accounted for only 18%. This ratio remained almost constant until 72 hpi. PBS injection provoked only mild fluctuations of this ratio.

The data show that infection activated very swiftly the cellular branch of innate immune defense by recruiting well-known effector cells and this occurred concomitantly and was conceivably triggered by the induced expression of key cytokines.

### 3.5. Significant Cell Migration of T and B Cells Is Likely to Occur in Head Kidney from 12 hpi Onwards

**Figure 6:**
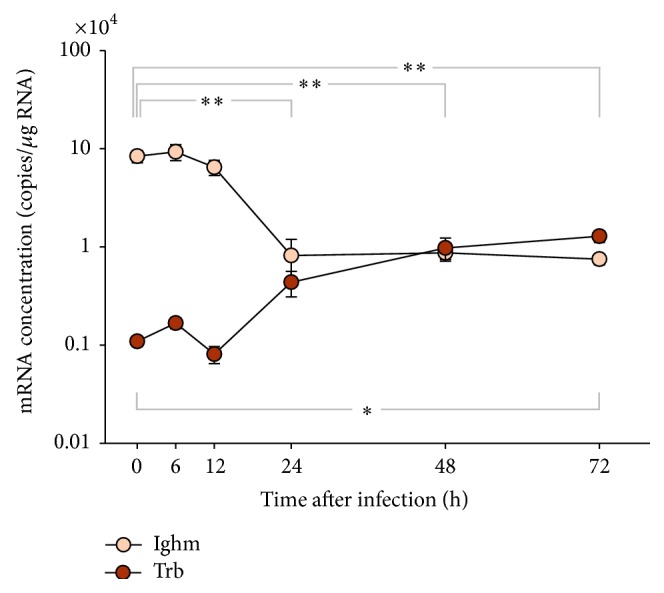
Dynamics of T and B cell migration in head kidney after infection. Ordinate shows the relative number of mRNA copies (per *μ*g total RNA) encoding the T cell receptor beta (TRB) and immunoglobulin M (Ighm) inhead kidney of five individuals/time point at various times after infection (abscissa). Values are given as mean ± SEM. Note that the relative quantity is presented on a log10 scale. Asterisks denote statistical significance with *p* < 0.01 (*∗∗*) and *p* < 0.05 (*∗*) compared to controls above (*ighm*) and below (*trb*) the graph. Statistical significance was assessed with a double sided *t*-test.

We profiled the dynamics of lymphoid cell migration in the four immune organs in order to validate the onset of adaptive immune activities involving T and B cells. T cell receptor *β* (*trb*) and immunoglobulin M, heavy chain (*ighm*) are broadly used gene markers indicating the presence of cells of the T- or B-type lineage [39]. We found only in the head kidney clear and significant changes of these markers (*p* = 0.03). The* trb* level remained stable for 12 hpi and subsequently rose to reach its maximum 12-fold increase at 72 hpi. Concomitantly, the* ighm* level dropped steeply after 12 hpi and reached a lower level plateau (11-fold reduction) at 24 hpi (Figure 6). This observation indicates either that B cells emigrated from the head kidney or that T or myeloid cells immigrated into this organ or that lymphocytes were strongly induced to proliferate here, thereby affecting the proportion of immune cells.

Changes of* trb *and* ighm* levels were all less than 3-fold and mostly statistically insignificant in the other three organs (thymus, spleen, and liver; Table 4).

## 4. Discussion

Toll-like receptors are key components of the innate immune system promoting a proinflammatory state after invasion of pathogens. Analyzing the tissue-specific and immune-modulated expression of a comprehensive set of factors contributing to TLR signal transduction may therefore inform about their role to overcome microbial threats (see [26] for a review). RT-qPCR is often the method of choice for the analysis due to the lack of fish-specific antibodies [26] and its superior sensitivity [40]. Moreover, a significant and positive correlation between the concentration of mRNA and its encoded protein has been found for a vast number of genes [41] and also for* tlr* genes in fish [42].

The prime interest of our study was to analyze in trout the role of TLR signaling to combat infection with the Gram-negative pathogen* A. salmonicida*, since no Tlr is known in teleost fish to recognize LPS, the major component of the outer cell wall of this pathogen. Prominent feature of our comprehensive survey of* tlr* expression during infection was that their expression was only moderately regulated, if at all. We conclude from this observation that* A. salmonicida* infection strongly induced proinflammatory mechanisms but failed to induce those pathways controlling the expression of genes encoding TLRs and associated factors. TLR expression is known to be regulated by the JAK/STAT signaling cascade [43] and hence our data hint, by inference, that also the latter signaling cascade was not largely activated by the infection. Beyond that, such comparatively small modulations in the levels of* tlr* transcripts cannot unequivocally be attributed to altered gene expression since they might as well reflect altered cell composition in the respective organs due to cell migration during the up-running immune defense. For example, large differences have recently been reported regarding the organ-specific content of* tlr*-expressing IgM^+^ cells in trout [44].

This general pattern of only a moderate regulation of* tlr *expression during infection in* O. mykiss* contrasts reports from other organisms. Pronounced upregulation of* tlr* expression after infection with relevant pathogens has been documented for mammals [21, 23], invertebrates [45, 46], and also bony fish. Distinct sets of* tlr* genes showed enhanced expression in channel catfish* Ictalurus punctatus *[19], Atlantic salmon* Salmo salar* [18], and Antarctic bullhead notothen* Notothenia coriiceps* [20] after parasitic, bacterial, and viral infection, respectively. After infection, some of those* tlr* genes revealed distinctively high mRNA abundances with changes of more than 10-fold above controls.

### 4.1. Tlr22a2 Was the Most Conspicuously Regulated* tlr* but Is Conceivably Not Specific for* A. salmonicida* Recognition

Tlr22 is important for induced cytokine synthesis [17]. The unique and quick sixfold upregulation of* tlr22a2* already 6 hpi in head kidney would highlight this TLR as a candidate for specifically contributing to* A. salmonicida* recognition. Yet, attributing that specific role to this factor is highly unlikely since the expression of the fish-specific* tlr22* is known to be modulated by a variety of apparently unrelated signals. It was significantly upregulated in several tissues and immune cells after infection and/or stimulation (i) with Gram-negative bacteria, that is,* Aeromonas* sp. in trout [47], in goldfish* Carassius auratus* [48], and in rohu* Labeo rohita* [49], as well as with* Vibrio anguillarum* in sea bream* Sparus aurata* [50]; (ii) with Gram-positive* Mycobacterium chelonae* in* C. auratus* [48]; (iii) with reovirus in grass carp* Ctenopharyngodon idella *[51, 52]; and (vi) with the ectoparasite* Argulus siamensis* in common carp* Cyprinus carpio* [53]. The broad spectrum of apparent “ligands” for this factor suggests that its interaction with MAMPs can structurally not be similar to the sophisticated key-lock principal as known from mammalian TLRs interacting with their specific ligands [54, 55]. Perhaps trout Tlr22a2 senses some endogenous DAMPs resulting from* Aeromonas*-induced traumata rather than exogenous MAMPs as previously discussed by Ingerslev et al. [14]. However, assuming ligand-independent activation of Tlr22a2 expression is even more puzzling since we noted in our original description of the twin receptors Tlr22a1 and Tlr22a2 [47] that both factors share a high degree of identical amino acid residues (94%), with most of the few exchanged residues being located in the N-terminal, distal leucine-rich repeat region (LRR). LRRs are known as ligand-binding areas of TLRs. We also note that the expression of both Tlr22a twin factors is not coregulated since only one of them was upregulated.

### 4.2. Irak1 May Have a Peculiar Role for Constraining* tlr* Signaling in Trout

Our candidate factors contributing downstream to* tlr* signaling were eventually upregulated in the livers of infected fish and none of them was significantly downregulated at any time after infection. Upregulated mRNA concentrations of such factors in liver were reported from a variety of challenge studies and infection experiments in different fish species [56–59]. Irak1 was distinct from all those other factors in so far as its mRNA abundance was increased already at 6 hpi, not only in liver, but also in spleen and head kidney. Early stimulation of the expression of this factor in several immune organs connects its activation to the very early events of mounting an immune defense. A prominent function of IRAK1 in mammals is its integration into the Myddosome, which is built up around the activated TIR domain of TLRs [8]. Viral infection of the grass carp was demonstrated to recruit Irak1 to the cell membrane possibly indicating that it is recruited to transmembrane TLRs [58]. Relating this information with our data could suggest that rate-limiting low levels of* irak1* factors might constrain TLR signaling in healthy trout and its organs. Given the absence of strong transcriptional* tlr* regulation in trout, this would conceptually allow shifting the regulatory level of the TLR signaling cascade away from the transcriptional regulation of some key receptors (such as* TLR2* and* TLR4* in mammals) towards the expression level of rate-limiting downstream factors.

Moreover, IRAK1 is also the factor being functionally inactivated by TOLLIP [58, 60]. Intriguingly enough, we found a consistent and significant upregulation of* tollip* expression in liver from 24 hpi onwards. This conceivably reflects the dampening of the synthesis of acute-phase factors. Hence, the data altogether allow the conclusion that Irak1 plays a prominent role in controlling the activity of TLR signaling in trout. Clearly, validation of this hypothesis would require a different experimental approach.

### 4.3. Trout Combats* A. salmonicida* Infection through Fast Recruitment of Myeloid Cells

Fast and strong recruitment of myeloid cells into the peritoneum indicates that the infected fish were prepared to combat the infectious pathogens. This almost instantaneous reaction against the invaders was in stark contrast to the sluggish modulation of the expression of factors contributing to the TLR signaling cascade. Recognizing the presence of pathogens by the host activated vibrantly the immune defense. The first step consisted of mounting a massive cellular defense to fight off the bacteria rather than inducing resilience or tolerance. Those myeloid cells (conceivably monocytes/macrophages and granulocytes), having been recruited into the peritoneum, belong to the cellular arm of innate immune defense. The second step of harnessing cellular immune mechanisms consisted of activating lymphoid cells, which belong to the adaptive branch of immune defense. The activation of adaptive immunity involves time-consuming processes such as induction of cell proliferation and cellular redifferentiation. Only then, significant amounts of cells might start migrating out from their site of proliferation and maturation to the site of infection. Our RT-qPCR data suggest belated onset of adaptive immunity, which was not surprising. It took more than 12 hpi for a considerable amount of the cells of the B-type lineage to probably move out from their reservoir in the head kidney [61] into the peritoneum. At the same time, the T lymphocytes presumably started moving into this organ, conceivably to be functionally primed by relevant antigen-presenting cells.

## 5. Conclusions

The presence of infectious* A. salmonicida* is quickly recognized by trout since the expression of key cytokine-encoding genes is instantaneously induced. However, even the severe infection with high doses of this Gram-negative pathogen only modestly regulated the expression of most* tlr* genes in immune organs indicating a mainly constitutive expression of these factors. We suggest that the limited abundance of Irak1 constrains their activity. No specific TLR could be singled out as a dominant receptor for perceiving the presence of* A. salmonicida*, albeit that Tlr22a2 was quickly and strongly induced after infection. Rather, this factor is known to be regulated by several nonrelated stimuli and might serve as unspecific alarm switch. The massive recruitment of granulocytes and monocytes/macrophages into the peritoneum highlights their pivotal importance. Hence, recruitment of cellular factors of innate immunity rather than induced expression of pathogen receptors is of key importance for mounting a first immune defense against invading* A. salmonicida*.

## Figures and Tables

**Table 1:** Oligonucleotide primers used in this study.

Primer name	Sense (3′-5′)	Antisense (3′-5′)	Primer efficiency	Fragment length [bp]	Tm [°C]	Accession code
TLR1_RT		TGGAAATCCTCACGGCCAAG				NM_001166101
TLR1_LC	AAAGACAGAGATGAGGGCTGTG	CGGTGCATGGAGGTAGGTTTC	2.04	161	85.5	
TLR2_RT		GTCATGGATGATAGAGGAGACGA				HE979560
TLR2_LC	GGAGAGGAGACCTGGCTGGA	CCTGGAATGGTCCTCGGAGA	1.88	141	85.6	
TLR3_RT		CCAGGCCTTTGAAGGTGGTG				NM_001124578
TLR3_LC	AGTCCTCCACCTGTCGAATCTA	GATCGCTGTCCCAGAAAGCTC	2.01	154	83.1	
TLR5_RT		TCCCCGTGCCCATATCATCT				NM_001124744
TLR5_LC	CAACTTCTTCGTTTGGCTGATAAT	ACCAGAGAAACTCAATGTGCATTA	1.94	160	78.7	
TLR7_RT		AGCTGTAAAAACGCTCCCTCCTC				GQ422119
TLR7_LC	TGCACAGAGAGTGTGACTATCAA	ATGTCAGAGAGGTTCGTTTTCAG	2.00	179	82.9	
TLR8a1_RT		GAAGGCACCTTCGGCAATGT				GQ422121
TLR8a1_LC	CTGTGTCACTTCCTGGTCATAAA	TTTTCTGATAGGTCCAGCACAGT	2.05	187	82.5	
TLR8a2_RT		GAGCATTGGAGCATCGTGGA				GQ422120
TLR8a2_LC	TCTCGAATATGAGCAACCTTGTC	AGTCCTTTTGGAACACGAGTTAAA	1.97	183	80.5	
TLR9_RT		TGCTCGTCAGCACAAACACG				NM_001129991
TLR9_LC	TGGTATTGCTTCCAGGTGCTGT	CTCCAGGTGGACCAGCAGCT	2.01	155	88.0	
TLR19_RT		TCTCCAGGCCACACCAGTCA				HF952170
TLR19_LC	GAGAGGAGGAGGTGAGGTATG	ATGCCCTTCCCTACCTCAAAGT	2.07	154	86.4	
TLR20_RT		GCCAGGGTGTGGTTCAGGAG				DQ872849
TLR20_LC	ACCCTCCGTCTGCTGGTGGA	CCACAGACGGTCCCAGAAGG	1.87	174	87.3	
TLR21_RT		TCTCCCAGGTCCAGGTAGCAA				CX261318
TLR21_LC	CTGTTGAGGCAGGTGCAACTAA	CTCAAAGGAGGTTTTTGGGAATC	2.06	156	83.2	
TLR22a1_RT		CGGGATAAATCCAACAGCCTCA				NM_001124412
TLR22a1_LC	TGGACAATGACGCTCTTTTACC	GAGCTGATGGTTGCAATGAGG	2.02	151	83.6	
TLR22a2_RT		TCCCAATGGATTCCGGGATAA				NM_001124419
TLR22a2_LC	TAGAAGTCGTACCAAAAGACATTC	GAGAGACCCATCATCCACTGAA	2.03	161	79.8	
NOD1_RT		Oligo(dT)				CA372055
NOD1_LC	CCGGTCAGCTGCTACGATGC	AGTGCAGCAGGAGGGGCAAAA	2.02	160	85.0	
MYD88_RT		CCCCCTTCTGGGTCATCCTC				NM_001124421
MYD88_LC	GATCAAGAATTACGAGGATTGCC	CCTCAATGAGAACTCGGAGATC	1.92	155	83.7	
IRAK4a_RT		TGAGGGATAGTCGTTCTTCCTGTTG				FN598575
IRAK4a_LC	GAGATTGATGAGGAGGAGATGG	TCAAGCTCTGTCAGGACCTCTT	2.02	161	84.7	
IRAK1_RT		CTGCGACGGAGCAGCAAGT				CA378286
IRAK1_LC	ATGGACAGTATCTCCGATGTGG	TGCAGCTTGTCCAGTACGTTCA	1.97	173	85.2	
TRAF6_RT		AAGCCCTTGGGGTTCCTCTG				BX081400
TRAF6_LC	CTCCAAACGCCCTCGGCACA	CCACATGGTGCTGGCCCTCA	1.84	154	88.4	
TOLLIP_RT		CAGTGGGCACTCCTGCAATG				NM_001124420
TOLLIP_LC	CTCAGTGGCAGACAAGGCGA	TCAGATATGGACATCTGCTTA	2.06	537	86.6	
IL1B_RT		TTCCACAGCACTCTCCAGCAA				NM_001124347
IL1B_LC	AAGTCTTTAAGCAACTGACTAAGC	TGCACTTTCAGAGGTGTTCTTTAT	2.06	152	79.2	
TNF_RT		CTGACCTTACCCCGCTAAGAAGA				NM_001124357
TNF_LC	GATACCCACCATACATTGAAGCA	ATTTGGTTCCCCTGTAGCTCGA	2.01	162	84.9	
IL8_RT		TTCCAACCTGGATAGAACATGATAGAA				AJ279069
IL8_LC	CTGAGGGGATGAGTCTGAGAG	ATCTCCTGACCGCTCTTGCTC	2.05	162	85.3	
IL10_RT		GGTTTGAACAACAATGCGCAGA				AB118099
IL10_LC	CTCAGATGCGGTTTTCAAACACT	CACAGTAAGCACTGGAACACAC	2.03	155	81.5	
TGFB_RT		CCTTGTGTTGTCTCCCCACATAAT				X99303
TGFB_LC	ATCAGGGATGAACAAGCTGAGG	TTCGCACACAGCAACTCTCCG	1.72	182	83.5	
IgM_RT		GAGCTGACTGTAGACAGAGTAG				S63348
IgM_LC	TACAAGAGGGAGACCGGAGGA	CACCGGCTCATCGTCAACAAG	1.92	159	84,5	
TRB_RT		ATGAAGATGCTGTAGGCCAGCT				EU072699
TRB_LC	GTCTTCTGGCAAGTCAACAATGT	GTAAAAGCTGACAATGCAGGTGA	1.94	165	83.7	

**Table 2:** Expression profiles of various cytokines in selected tissues of rainbow trout at different time points after infection.

Gene symbol	Tissues	Fold change values at time points after infection relative to control				
		6 h	12 h	24 h	48 h	72 h
*il1b *	Spleen	**39.35 ±** 12.98^*∗*^	**47.80 ±** 13.94^*∗*^	7.54 ± 2.33	8.89 ± 2.64	3.63 ± 1.04
	Head kidney	180.12 ± 75.62	**393.18 ±** 95.32^*∗*^	17.50 ± 7.97	17.39 ± 5.72	28.38 ± 8.11
	Liver	68.47 ± 26.01	**151.60 ±** 42.63^*∗*^	19.35 ± 6.24	22.21 ± 8.50	81.23 ± 32.69
	Thymus	3.36 ± 0.73	**9.74 ±** 2.93^*∗*^	2.00 ± 0.57	1.63 ± 0.35	2.75 ± 0.66

*tnf *	Spleen	8.68 ± 4.55	**7.76 ±** 2.34^*∗*^	−1.16 ± 0.37	1.12 ± 0.30	1.82 ± 0.52
	Head kidney	2.06 ± 0.70	**11.94 ±** 4.32^*∗*^	−1.18 ± 0.53	−1.31 ± 0.26	1.64 ± 0.38
	Liver	**21.10 ±** 13.46^*∗*^	**11.58 ±** 4.93^*∗*^	3.74 ± 1.68	8.50 ± 4.33	10.88 ± 5.68
	Thymus	1.18 ± 0.28	2.10 ± 0.74	−1.27 ± 0.36	−1.13 ± 0.15	1.54 ± 0.25

*il8 *	Spleen	**27.85 ±** 11.11^*∗*^	14.92 ± 4.55	4.30 ± 1.31	13.37 ± 6.27	**17.80 ±** 6.48^*∗*^
	Head kidney	**26.59 ±** 9.13^*∗*^	19.11 ± 5.15	7.24 ± 1.89	13.14 ± 4.92	**24.12 ±** 7.00^*∗*^
	Liver	**60.14 ±** 24.25^*∗*^	25.32 ± 13.09	9.30 ± 3.11	71.70 ± 55.32	**39.32 ±** 22.19^*∗*^
	Thymus	1.13 ± 0.31	1.33 ± 0.48	−1.38 ± 0.35	−1.14 ± 0.52	1.25 ± 0.28

*il10 *	Spleen	1.24 ± 0.33	1.26 ± 0.27	1.46 ± 0.41	1.19 ± 0.35	1.29 ± 0.38
	Head kidney	20.39 ± 9.00	**54.04 ±** 18.64^*∗*^	10.35 ± 4.94	11.83 ± 6.24	15.53 ± 6.49
	Liver	2.52 ± 0.88	3.19 ± 1.12	3.08 ± 0.84	3.20 ± 0.95	**3.63 ± **1.14^*∗*^
	Thymus	1.51 ± 0.52	1.59 ± 0.50	1.41 ± 0.47	1.41 ± 0.38	1.15 ± 0.32

*tgfb *	Spleen	1.11 ± 0.08	−1.05 ± 0.09	1.14 ± 0.15	1.04 ± 0.11	−1.06 ± 0.12
	Head kidney	1.17 ± 0.12	1.10 ± 0.09	1.14 ± 0.12	1.06 ± 0.14	−1.11 ± 0.13
	Liver	1.34 ± 0.14	1.50 ± 0.18	**2.17 **± 0.35^*∗*^	1.76 ± 0.24	1.50 ± 0.20
	Thymus	−1.13 ± 0.16	−1.40 ± 0.22	−1.42 ± 0.33	−1.21 ± 0.24	−1.06 ± 0.16

^*∗*^Significant expression difference (*p* < 0.05) between the infected and the control groups is indicated in bold.

**(a):** 

Gene symbol	Tissues	Fold change values at time points after infection relative to control				
		6 h	12 h	24 h	48 h	72 h
*tlr1 *	Spleen	−1.07 ± 0.19	−1.02 ± 0.23	1.07 ± 0.16	−1.07 ± 0.18	−1.54 ± 0.28
	Head kidney	1.61 ± 0.18	1.72 ± 0.25	1.50 ± 0.27	1.57 ± 0.21	1.66 ± 0.13
	Liver	−1.11 ± 0.18	1.72 ± 0.31	2.32 ± 0.56	1.67 ± 0.31	1.70 ± 0.22
	Thymus	−1.31 ± 0.29	−1.20 ± 0.32	1.21 ± 0.29	1.13 ± 0.24	1.09 ± 0.20

*tlr2 *	Spleen	−1.13 ± 0.16	**−1.73 ± **0.33^*∗*^	**−1.86 ± **0.41^*∗*^	−1.46 ± 0.15	**−1.68 ± **0.20^*∗*^
	Head kidney	1.24 ± 0.19	−1.13 ± 0.17	−1.68 ± 0.28	−1.10 ± 0.22	−1.18 ± 0.15
	Liver	−1.01 ± 0.11	−1.11 ± 0.11	−1.01 ± 0.12	1.50 ± 0.32	1.16 ± 0.18
	Thymus	−1.12 ± 0.21	−1.07 ± 0.19	−1.07 ± 0.25	1.16 ± 0.19	1.22 ± 0.16

* tlr3 *	Spleen	−1.58 ± 0.17	−1.67 ± 0.25	−1.21 ± 0.25	**−1.82 ± **0.23^*∗*^	**−1.70 ± **0.23^*∗*^
	Head kidney	**−1.52 ± **0.17^*∗*^	**−1.99 ± **0.26^*∗*^	**−2.49 ± **0.30^*∗*^	**−2.08 ± **0.29^*∗*^	**−2.09 ± **0.32^*∗*^
	Liver	−1.21 ± 0.31	−1.64 ± 0.39	−1.40 ± 0.36	−1.83 ± 0.47	−1.64 ± 0.51
	Thymus	−1.26 ± 0.27	−1.87 ± 0.49	−1.46 ± 0.37	−1.52 ± 0.37	−1.63 ± 0.34

*tlr5 *	Spleen	−2.77 ± 0.71	**−3.88 ± **0.71^*∗*^	−2.76 ± 0.70	−2.34 ± 0.66	−1.61 ± 0.49
	Head kidney	−1.07 ± 0.27	1.03 ± 0.64	−2.33 ± 0.48	−1.62 ± 0.47	−1.55 ± 0.34
	Liver	1.28 ± 0.31	−1.30 ± 0.25	1.77 ± 0.37	1.99 ± 0.37	1.96 ± 0.38
	Thymus	1.14 ± 0.23	−1.12 ± 0.29	−2.08 ± 0.35	−1.29 ± 0.30	−1.91 ± 0.52

*tlr7 *	Spleen	**−2.10 ± **0.24^*∗*^	−1.62 ± 0.58	**−2.79 ± **0.48^*∗*^	**−3.23** ± 0.62^*∗*^	**−3.40 ± **0.85^*∗*^
	Head kidney	1.95 ± 0.45	2.22 ± 0.63	1.51 ± 0.49	−1.28 ± 0.35	−1.04 ± 0.24
	Liver	−1.01 ± 0.23	1.06 ± 0.19	1.17 ± 0.20	1.33 ± 0.41	−1.05 ± 0.30
	Thymus	−1.30 ± 0.28	−1.34 ± 0.33	−1.03 ± 0.40	−1.35 ± 0.28	−1.47 ± 0.31

*tlr8a1 *	Spleen	**−2.03 ± **0.29^*∗*^	**−1.76 ± **0.28^*∗*^	−1.29 ± 0.20	−1.36 ± 0.23	**−1.62 ± **0.29^*∗*^
	Head kidney	−1.35 ± 0.22	−1.89 ± 0.39	−1.90 ± 0.34	−1.30 ± 0.45	−1.89 ± 0.77
	Liver	1.47 ± 0.41	1.81 ± 0.42	2.86 ± 0.77	2.88 ± 1.00	1.67 ± 0.43
	Thymus	1.02 ± 0.21	−1.01 ± 0.27	−1.09 ± 0.28	1.15 ± 0.28	−1.41 ± 0.30

*tlr8a2 *	Spleen	**−2.45 ± **0.53^*∗*^	**−1.99 ± **0.29^*∗*^	−1.49 ± 0.27	**−1.85 ± **0.32^*∗*^	**−2.75 ± **0.45^*∗*^
	Head kidney	−1.31 ± 0.43	−1.46 ± 0.31	−1.23 ± 0.24	−1.29 ± 0.27	−1.91 ± 0.31
	Liver	1.54 ± 0.50	1.75 ± 0.56	2.30 ± 0.56	2.56 ± 0.81	1.54 ± 0.42
	Thymus	1.07 ± 0.27	−1.49 ± 0.33	−1.27 ± 0.32	1.03 ± 0.45	−1.28 ± 0.47

*tlr9 *	Spleen	1.09 ± 0.11	1.32 ± 0.11	1.44 ± 0.17	1.20 ± 0.22	**−2.00 ± **0.25^*∗*^
	Head kidney	1.33 ± 0.19	1.35 ± 0.25	1.24 ± 0.21	1.29 ± 0.18	1.43 ± 0.17
	Liver	1.50 ± 0.22	1.95 ± 0.32	2.57 ± 0.42	**2.87 ± **0.52^*∗*^	**3.44 ± **0.51^*∗*^
	Thymus	−1.22 ± 0.37	−1.20 ± 0.40	1.15 ± 0.26	1.20 ± 0.39	1.33 ± 0.42

*tlr19 *	Spleen	−1.22 ± 0.28	−1.66 ± 0.59	−1.62 ± 0.46	1.45 ± 0.38	−2.82 ± 0.66
	Head kidney	**3.18 ± **0.84^*∗*^	2.52 ± 0.54	2.73 ± 0.85	2.19 ± 0.64	2.00 ± 0.55
	Liver	2.83 ± 1.53	−1.82 ± 0.59	1.00 ± 0.23	−1.90 ± 0.54	−2.00 ± 0.57
	Thymus	1.06 ± 0.39	−1.22 ± 0.37	1.35 ± 0.55	1.02 ± 0.30	1.42 ± 0.43

*tlr20 *	Spleen	−1.58 ± 0.29	−1.71 ± 0.38	−1.23 ± 0.21	1.08 ± 0.27	−1.02 ± 0.25
	Head kidney	1.29 ± 0.20	−1.40 ± 0.21	1.07 ± 0.17	1.24 ± 0.18	1.49 ± 0.21
	Liver	1.55 ± 0.23	1.74 ± 0.20	**2.01 ± **0.19^*∗*^	**2.20 ± **0.25^*∗*^	**2.02 ± **0.26^*∗*^
	Thymus	−1.17 ± 0.24	1.32 ± 0.35	−1.22 ± 0.32	1.19 ± 0.26	1.42 ± 0.31

*tlr21 *	Spleen	−1.39 ± 0.28	**−3.82 ± **0.46^*∗*^	−1.83 ± 0.40	−1.25 ± 0.35	−1.03 ± 0.19
	Head kidney	−1.06 ± 0.26	**−2.96 ± **0.61^*∗*^	**−3.36 ± **0.51^*∗*^	−1.65 ± 0.26	−1.52 ± 0.24
	Liver	1.67 ± 0.48	1.61 ± 0.44	1.34 ± 0.21	2.16 ± 0.58	1.51 ± 0.34
	Thymus	1.07 ± 0.18	−1.31 ± 0.26	−1.47 ± 0.29	1.00 ± 0.21	−1.13 ± 0.21

*tlr22a1 *	Spleen	1.21 ± 0.27	−1.18 ± 0.22	−1.10 ± 0.23	1.01 ± 0.27	−1.99 ± 0.45
	Head kidney	1.55 ± 0.50	1.41 ± 0.34	1.51 ± 0.56	1.06 ± 0.24	−1.75 ± 0.44
	Liver	2.12 ± 0.52	1.94 ± 0.43	2.50 ± 0.58	3.23 ± 1.00	1.41 ± 0.34
	Thymus	1.24 ± 0.21	−1.11 ± 0.29	1.11 ± 0.35	1.07 ± 0.23	−1.46 ± 0.23

*tlr22a2 *	Spleen	3.02 ± 0.64	1.21 ± 0.24	1.11 ± 0.11	1.89 ± 0.39	1.53 ± 0.30
	Head kidney	**6.14 ± **2.37^*∗*^	**3.81 ± **0.70^*∗*^	1.63 ± 0.32	3.27 ± 0.70	2.71 ± 0.48
	Liver	3.51 ± 0.79	**4.41 ± **0.97^*∗*^	2.94 ± 0.85	**3.85 ± **0.97^*∗*^	3.12 ± 0.80
	Thymus	1.37 ± 0.25	1.83 ± 0.33	1.48 ± 0.40	1.23 ± 0.34	1.15 ± 0.28

*nod1 *	Spleen	−1.05 ± 0.15	−1.60 ± 0.20	−1.01 ± 0.14	1.07 ± 0.12	−1.13 ± 0.13
	Head kidney	−1.22 ± 0.23	**−3.08 ± **0.61^*∗*^	−1.60 ± 0.26	−1.31 ± 0.32	−1.59 ± 0.35
	Liver	1.20 ± 0.18	1.26 ± 0.19	1.37 ± 0.19	1.36 ± 0.30	1.74 ± 0.34
	Thymus	−1.02 ± 0.21	1.45 ± 0.33	1.35 ± 0.39	−1.04 ± 0.27	−1.15 ± 0.26

^*∗*^Significant expression difference (*p* < 0.05) between the infected and the control groups is indicated in bold.

**(b):** 

Gene symbol	Tissues	Fold change values of infected group relative to the control				
		6 h	12 h	24 h	48 h	72 h
*myd88 *	Spleen	1.44 ± 0.25	1.68 ± 0.24	1.33 ± 0.33	1.36 ± 0.22	1.25 ± 0.20
	Head kidney	1.37 ± 0.16	1.33 ± 0.20	1.22 ± 0.22	1.45 ± 0.29	1.04 ± 0.12
	Liver	2.75 ± 0.48	**6.68 ± **0.63^*∗*^	3.34 ± 0.76	4.37 ± 1.48	**6.78 ± **1.89^*∗*^
	Thymus	1.08 ± 0.14	−1.13 ± 0.23	1.01 ± 0.21	1.09 ± 0.17	1.00 ± 0.14

*irak4a *	Spleen	−1.02 ± 0.14	−1.20 ± 0.10	1.26 ± 0.09	1.03 ± 0.16	−1.06 ± 0.14
	Head kidney	1.04 ± 0.11	−1.25 ± 0.16	−1.36 ± 0.19	−1.31 ± 0.18	−1.39 ± 0.19
	Liver	1.38 ± 0.14	1.67 ± 0.14	**1.87 ± **0.24^*∗*^	1.51 ± 0.15	**1.86 ± **0.23^*∗*^
	Thymus	1.17 ± 0.16	1.10 ± 0.18	1.08 ± 0.18	1.26 ± 0.17	**1.71 ± **0.23^*∗*^

*irak1 *	Spleen	**2.81 ± **0.43^*∗*^	**2.86 ± **0.24^*∗*^	1.56 ± 0.17	1.51 ± 0.22	1.13 ± 0.20
	Head kidney	**2.24 ± **0.35^*∗*^	**2.54 ± **0.47^*∗*^	1.23 ± 0.15	1.34 ± 0.17	1.55 ± 0.18
	Liver	**2.60 ± **0.50^*∗*^	**3.93 ± **0.49^*∗*^	2.12 ± 0.35	2.14 ± 0.30	**2.34 ± **0.34^*∗*^
	Thymus	1.11 ± 0.13	−1.07 ± 0.19	−1.25 ± 0.21	−1.14 ± 0.16	1.13 ± 0.15

*traf6 *	Spleen	1.10 ± 0.15	1.29 ± 0.13	1.26 ± 0.13	1.21 ± 0.16	1.15 ± 0.13
	Head kidney	1.05 ± 0.09	1.49 ± 0.15	1.36 ± 0.14	**1.57 ± **0.15^*∗*^	1.37 ± 0.15
	Liver	1.27 ± 0.09	**2.21** ± 0.27^*∗*^	**2.21 ± **0.30^*∗*^	**2.04 ± **0.33^*∗*^	**2.17 ± **0.25^*∗*^
	Thymus	1.18 ± 0.15	−1.04 ± 0.11	−1.01 ± 0.15	−1.03 ± 0.13	**1.78 ± **0.22^*∗*^

*tollip *	Spleen	1.06 ± 0.19	1.21 ± 0.21	1.29 ± 0.22	1.15 ± 0.23	1.24 ± 0.21
	Head kidney	1.31 ± 0.21	−1.05 ± 0.24	1.14 ± 0.12	1.27 ± 0.14	1.11 ± 0.10
	Liver	1.62 ± 0.48	1.73 ± 0.54	2.74 ± 0.87	**3.44 ± **1.14^*∗*^	**4.53 ± **1.43^*∗*^
	Thymus	1.03 ± 0.15	−1.11 ± 0.29	1.05 ± 0.24	−1.04 ± 0.19	1.29 ± 0.26

^*∗*^Significant expression difference (*p* < 0.05) between the infected and the control groups is indicated in bold.

**Table 4:** Expression profiles of T and B cell marker genes in selected tissues of rainbow trout at different time points after infection.

Gene symbol	Tissues	Fold change values at time points after infection relative to control				
		6 h	12 h	24 h	48 h	72 h
*trb *	Spleen	−1.55 ± 0.27	**−2.00 ± **0.34^*∗*^	**−1.95 ± **0.64^*∗*^	−1.66 ± 0.27	**−2.76 ± **0.45^*∗*^
	Head kidney	1.53 ± 0.26	−1.35 ± 0.31	4.01 ± 1.25	8.94 ± 2.59	**11.78 ± **2.60
	Liver	−1.10 ± 0.40	−1.01 ± 0.35	2.80 ± 1.82	−1.50 ± 0.57	−1.47 ± 0.57
	Thymus	−1.05 ± 0.26	−1.48 ± 0.39	**−2.78 ± **0.87	−1.42 ± 0.37	−1.23 ± 0.28

*igm *	Spleen	−1.26 ± 0.31	−1.51 ± 0.33	−1.37 ± 0.34	1.09 ± 0.33	−1.67 ± 0.45
	Head kidney	1.11 ± 0.26	−1.30 ± 0.29	**−10.26 ± **4.95^*∗*^	**−9.64 ± **1.96^*∗*^	**−11.19 ± **2.19^*∗*^
	Liver	1.01 ± 0.35	−1.24 ± 0.42	2.26 ± 0.81	1.43 ± 0.52	1.43 ± 0.48
	Thymus	1.23 ± 0.28	1.06 ± 0.36	**2.92 ± **1.08^*∗*^	2.42 ± 0.54	2.42 ± 0.45

^*∗*^Significant expression difference (*p* < 0.05) between the infected and the control groups is indicated in bold.

## Abbreviations

hpi: Hours postinfection
Ig: Immunoglobulin
IL: Interleukin
IRAK: Interleukin-1 receptor-associated kinase
LRR: Leucine-rich repeat region
MYD88: Myeloid differentiation primary response protein 88
NOD1: Nucleotide-binding oligomerization domain-containing protein 1
PBS: Phosphate-buffered saline
RT-qPCR: Quantitative real-time reverse transcription polymerase chain reaction
TGFB: Transforming growth factor beta
TIR: Toll/interleukin-1 receptor domain
TLR: Toll-like receptor
TNF: Tumor necrosis factor (alpha)
TOLLIP: Toll-interacting protein
TRAF6: TNF receptor-associated factor 6
TRB: T cell receptor beta locus.

## References

[1] Rauta P. R., Nayak B., Das S. (2012). Immune system and immune responses in fish and their role in comparative immunity study: a model for higher organisms. *Immunology Letters*.

[2] Bianchi M. E. (2007). DAMPs, PAMPs and alarmins: all we need to know about danger. *Journal of Leukocyte Biology*.

[3] Kaczmarek A., Vandenabeele P., Krysko D. V. (2013). Necroptosis: the release of damage-associated molecular patterns and its physiological relevance. *Immunity*.

[4] Gill R., Tsung A., Billiar T. (2010). Linking oxidative stress to inflammation: toll-like receptors. *Free Radical Biology & Medicine*.

[5] Zhang J., Kong X., Zhou C., Li L., Nie G., Li X. (2014). Toll-like receptor recognition of bacteria in fish: ligand specificity and signal pathways. *Fish and Shellfish Immunology*.

[6] Rebl A., Goldammer T., Seyfert H.-M. (2010). Toll-like receptor signaling in bony fish. *Veterinary Immunology and Immunopathology*.

[7] Roach J. C., Glusman G., Rowen L. (2005). The evolution of vertebrate Toll-like receptors. *Proceedings of the National Academy of Sciences of the United States of America*.

[8] Lin S.-C., Lo Y.-C., Wu H. (2010). Helical assembly in the MyD88-IRAK4-IRAK2 complex in TLR/IL-1R signalling. *Nature*.

[9] Brietzke A., Goldammer T., Rebl H. (2014). Characterization of the interleukin 1 receptor-associated kinase 4 (IRAK4)-encoding gene in salmonid fish: the functional copy is rearranged in *Oncorhynchus mykiss* and that factor can impair TLR signaling in mammalian cells. *Fish and Shellfish Immunology*.

[10] Kawai T., Akira S. (2008). Toll-like receptor and RIG-1-like receptor signaling. *Annals of the New York Academy of Sciences*.

[11] Belvin M. P., Anderson K. V. (1996). A conserved signaling pathway: the *Drosophila* toll-dorsal pathway. *Annual Review of Cell and Developmental Biology*.

[12] Patterson N. J., Werling D. (2013). To con protection: TIR-domain containing proteins (Tcp) and innate immune evasion. *Veterinary Immunology and Immunopathology*.

[13] Daly J. G., Kew A. K., Moore A. R., Olivier G. (1996). The cell surface of *Aeromonas salmonicida* determines *in vitro* survival in cultured brook trout (*Salvelinus fontinalis*) peritoneal macrophages. *Microbial Pathogenesis*.

[14] Ingerslev H. C., Lunder T., Nielsen M. E. (2010). Inflammatory and regenerative responses in salmonids following mechanical tissue damage and natural infection. *Fish & Shellfish Immunology*.

[15] Robertsen B. (2006). The interferon system of teleost fish. *Fish & Shellfish Immunology*.

[16] Hansen J. D., Vojtech L. N., Laing K. J. (2011). Sensing disease and danger: a survey of vertebrate PRRs and their origins. *Developmental and Comparative Immunology*.

[17] Matsuo A., Oshiumi H., Tsujita T. (2008). Teleost TLR22 recognizes RNA duplex to induce IFN and protect cells from birnaviruses. *Journal of Immunology*.

[18] Arnemo M., Kavaliauskis A., Gjøen T. (2014). Effects of TLR agonists and viral infection on cytokine and TLR expression in Atlantic salmon (*Salmo salar*). *Developmental and Comparative Immunology*.

[19] Zhao F., Li Y.-W., Pan H.-J. (2013). Expression profiles of toll-like receptors in channel catfish (*Ictalurus punctatus*) after infection with *Ichthyophthirius multifiliis*. *Fish & Shellfish Immunology*.

[20] Ahn D. H., Shin S. C., Park H. (2014). Characterization of Toll-like receptor gene expression and the pathogen agonist response in the antarctic bullhead notothen *Notothenia coriiceps*. *Immunogenetics*.

[21] Fuller C. L., Brittingham K. C., Porter M. W. (2007). Transcriptome analysis of human immune responses following live vaccine strain (LVS) *Francisella tularensis* vaccination. *Molecular Immunology*.

[22] Tsujimoto H., Ono S., Majima T. (2005). Neutrophil elastase, MIP-2, and TLR-4 expression during human and experimental sepsis. *Shock*.

[23] Okabayashi T., Kariwa H., Yokota S.-I. (2006). Cytokine regulation in SARS coronavirus infection compared to other respiratory virus infections. *Journal of Medical Virology*.

[24] Beutler B., Rietschel E. T. (2003). Innate immune sensing and its roots: the story of endotoxin. *Nature Reviews Immunology*.

[25] Iliev D. B., Roach J. C., Mackenzie S., Planas J. V., Goetz F. W. (2005). Endotoxin recognition: in fish or not in fish?. *FEBS Letters*.

[26] Pietretti D., Wiegertjes G. F. (2014). Ligand specificities of Toll-like receptors in fish: indications from infection studies. *Developmental and Comparative Immunology*.

[27] Rebl A., Goldammer T., Fischer U., Köllner B., Seyfert H.-M. (2009). Characterization of two key molecules of teleost innate immunity from rainbow trout (*Oncorhynchus mykiss*): MyD88 and SAA. *Veterinary Immunology and Immunopathology*.

[28] Rebl A., Høyheim B., Fischer U., Köllner B., Siegl E., Seyfert H.-M. (2008). Tollip, a negative regulator of TLR-signalling, is encoded by twin genes in salmonid fish. *Fish and Shellfish Immunology*.

[29] Korytář T., Jaros J., Verleih M. (2013). Novel insights into the peritoneal inflammation of rainbow trout (*Oncorhynchus mykiss*). *Fish and Shellfish Immunology*.

[30] Park S. B., Hikima J.-I., Suzuki Y. (2012). Molecular cloning and functional analysis of nucleotide-binding oligomerization domain 1 (NOD1) in olive flounder, *Paralichthys olivaceus*. *Developmental and Comparative Immunology*.

[31] Zou J., Grabowski P. S., Cunningham C., Secombes C. J. (1999). Molecular cloning of interleukin 1*β* from rainbow trout *Oncorhynchus mykiss* reveals no evidence of an ice cut site. *Cytokine*.

[32] Pleguezuelos O., Zou J., Cunningham C., Secombes C. J. (2000). Cloning, sequencing, and analysis of expression of a second IL-1beta gene in rainbow trout (*Oncorhynchus mykiss*). *Immunogenetics*.

[33] Laing K. J., Wang T., Zou J. (2001). Cloning and expression analysis of rainbow trout Oncorhynchus mykiss tumour necrosis factor-*α*. *European Journal of Biochemistry*.

[34] Laing K. J., Zou J. J., Wang T. H. (2002). Identification and analysis of an interleukin 8-like molecule in rainbow trout *Oncorhynchus mykiss*. *Developmental and Comparative Immunology*.

[35] Rebl A., Rebl H., Korytář T., Goldammer T., Seyfert H.-M. (2014). The proximal promoter of a novel interleukin-8-encoding gene in rainbow trout (*Oncorhynchus mykiss*) is strongly induced by CEBPA, but not NF-*κ*B p65. *Developmental and Comparative Immunology*.

[36] Inoue Y., Kamota S., Ito K. (2005). Molecular cloning and expression analysis of rainbow trout (*Oncorhynchus mykiss*) interleukin-10 cDNAs. *Fish and Shellfish Immunology*.

[37] Hardie L. J., Laing K. J., Daniels G. D., Grabowski P. S., Cunningham C., Secombes C. J. (1998). Isolation of the first piscine transforming growth factor *β* gene: analysis reveals tissue specific expression and a potential regulatory sequence in rainbow trout (*Oncorhynchus mykiss*). *Cytokine*.

[38] Sabat R., Grütz G., Warszawska K. (2010). Biology of interleukin-10. *Cytokine & Growth Factor Reviews*.

[39] Bernard D., Six A., Rigottier-Gois L. (2006). Phenotypic and functional similarity of gut intraepithelial and systemic T cells in a teleost fish. *Journal of Immunology*.

[40] Visintin A., Mazzoni A., Spitzer J. H., Wyllie D. H., Dower S. K., Segal D. M. (2001). Regulation of Toll-like receptors in human monocytes and dendritic cells. *Journal of Immunology*.

[41] Schwanhäusser B., Busse D., Li N. (2011). Global quantification of mammalian gene expression control. *Nature*.

[42] Skjæveland I., Iliev D. B., Zou J., Jørgensen T., Jørgensen J. B. (2008). A TLR9 homolog that is up-regulated by IFN-*γ* in Atlantic salmon (*Salmo salar*). *Developmental and Comparative Immunology*.

[43] Hu X., Chen J., Wang L., Ivashkiv L. B. (2007). Crosstalk among Jak-STAT, Toll-like receptor, and ITAM-dependent pathways in macrophage activation. *Journal of Leukocyte Biology*.

[44] Abós B., Castro R., Pignatelli J., Luque A., González L., Tafalla C. (2013). Transcriptional heterogeneity of IgM^+^ cells in rainbow trout (*Oncorhynchus mykiss*) tissues. *PLoS ONE*.

[45] Deepika A., Sreedharan K., Paria A., Makesh M., Rajendran K. V. (2014). Toll-pathway in tiger shrimp (*Penaeus monodon*) responds to white spot syndrome virus infection: evidence through molecular characterisation and expression profiles of MyD88, TRAF6 and TLR genes. *Fish and Shellfish Immunology*.

[46] Wang P.-H., Liang J.-P., Gu Z.-H. (2012). Molecular cloning, characterization and expression analysis of two novel Tolls (LvToll2 and LvToll3) and three putative Spätzle-like Toll ligands (LvSpz1-3) from *Litopenaeus vannamei*. *Developmental and Comparative Immunology*.

[47] Rebl A., Siegl E., Köllner B., Fischer U., Seyfert H.-M. (2007). Characterization of twin toll-like receptors from rainbow trout (*Oncorhynchus mykiss*): evolutionary relationship and induced expression by *Aeromonas salmonicida salmonicida*. *Developmental and Comparative Immunology*.

[48] Stafford J. L., Ellestad K. K., Magor K. E., Belosevic M., Magor B. G. (2003). A toll-like receptor (TLR) gene that is up-regulated in activated goldfish macrophages. *Developmental and Comparative Immunology*.

[49] Samanta M., Swain B., Basu M. (2014). Toll-like receptor 22 in *Labeo rohita*: molecular cloning, characterization, 3D modeling, and expression analysis following ligands stimulation and bacterial infection. *Applied Biochemistry and Biotechnology*.

[50] Muñoz I., Sepulcre M. P., Meseguer J., Mulero V. (2014). Toll-like receptor 22 of gilthead seabream, *Sparus aurata*: molecular cloning, expression profiles and post-transcriptional regulation. *Developmental and Comparative Immunology*.

[51] Lv J., Huang R., Li H. (2012). Cloning and characterization of the grass carp (*Ctenopharyngodon idella*) Toll-like receptor 22 gene, a fish-specific gene. *Fish & Shellfish Immunology*.

[52] Rao Y., Su J. (2015). Insights into the antiviral immunity against grass carp (*Ctenopharyngodon idella*) reovirus (GCRV) in grass carp. *Journal of Immunology Research*.

[53] Panda R. P., Chakrapani V., Patra S. K. (2014). First evidence of comparative responses of Toll-like receptor 22 (TLR22) to relatively resistant and susceptible Indian farmed carps to *Argulus siamensis* infection. *Developmental and Comparative Immunology*.

[54] Park B. S., Song D. H., Kim H. M., Choi B.-S., Lee H., Lee J.-O. (2009). The structural basis of lipopolysaccharide recognition by the TLR4-MD-2 complex. *Nature*.

[55] Manavalan B., Basith S., Choi S. (2011). Similar structures but different roles—an updated perspective on TLR structures. *Frontiers in Physiology*.

[56] Wei J., Guo M., Gao P. (2014). Isolation and characterization of tumor necrosis factor receptor-associated factor 6 (TRAF6) from grouper, *Epinephelus tauvina*. *Fish & Shellfish Immunology*.

[57] Yu Y., Zhong Q., Li C. (2012). Identification and characterization of IL-1 receptor-associated kinase-4 (IRAK-4) in half-smooth tongue sole *Cynoglossus semilaevis*. *Fish & Shellfish Immunology*.

[58] Huang R., Lv J., Luo D., Liao L., Zhu Z., Wang Y. (2012). Identification, characterization and the interaction of Tollip and IRAK-1 in grass carp (*Ctenopharyngodon idellus*). *Fish and Shellfish Immunology*.

[59] Zhang C.-Z., Yin Z.-X., He W. (2009). Cloning of IRAK1 and its upregulation in symptomatic mandarin fish infected with ISKNV. *Biochemical and Biophysical Research Communications*.

[60] Bulut Y., Faure E., Thomas L., Equils O., Arditi M. (2001). Cooperation of Toll-like receptor 2 and 6 for cellular activation by soluble tuberculosis factor and *Borrelia burgdorferi* outer surface protein a lipoprotein: role of Toll-interacting protein and IL-1 receptor signaling molecules in Toll-like receptor 2 signaling. *The Journal of Immunology*.

[61] Hansen J. D., Zapata A. G. (1998). Lymphocyte development in fish and amphibians. *Immunological Reviews*.

